# Engaging ethnic minority communities through performance and arts: health education in Cambodian forest villages

**DOI:** 10.1093/inthealth/ihaa076

**Published:** 2020-10-10

**Authors:** James J Callery, Nou Sanann, Rupam Tripura, Thoek Buntau, Thomas J Peto, Pich Kunthea, Christopher Pell, Ung Soviet, Chea Nguon, Dysoley Lek, Phaik Yeong Cheah

**Affiliations:** Mahidol Oxford Tropical Medicine Research Unit, Faculty of Tropical Medicine, Mahidol University, Bangkok, Thailand; Mahidol Oxford Tropical Medicine Research Unit, Faculty of Tropical Medicine, Mahidol University, Bangkok, Thailand; University Research Company, Phnom Penh, Cambodia; Mahidol Oxford Tropical Medicine Research Unit, Faculty of Tropical Medicine, Mahidol University, Bangkok, Thailand; Centre for Tropical Medicine and Global Health, Nuffield Department of Clinical Medicine, University of Oxford, Oxford, UK; Mahidol Oxford Tropical Medicine Research Unit, Faculty of Tropical Medicine, Mahidol University, Bangkok, Thailand; Mahidol Oxford Tropical Medicine Research Unit, Faculty of Tropical Medicine, Mahidol University, Bangkok, Thailand; Centre for Tropical Medicine and Global Health, Nuffield Department of Clinical Medicine, University of Oxford, Oxford, UK; Mahidol Oxford Tropical Medicine Research Unit, Faculty of Tropical Medicine, Mahidol University, Bangkok, Thailand; Amsterdam Institute for Global Health and Development, Amsterdam, The Netherlands; Centre for Social Sciences and Global Health, University of Amsterdam, Amsterdam, The Netherlands; Provincial Health Department of Stung Treng Province, Cambodia; National Center for Parasitology, Entomology and Malaria Control, Phnom Penh, Cambodia; National Center for Parasitology, Entomology and Malaria Control, Phnom Penh, Cambodia; School of Public Health, National Institute of Public Health, Phnom Penh, Cambodia; Mahidol Oxford Tropical Medicine Research Unit, Faculty of Tropical Medicine, Mahidol University, Bangkok, Thailand; Centre for Tropical Medicine and Global Health, Nuffield Department of Clinical Medicine, University of Oxford, Oxford, UK

**Keywords:** Cambodia, community engagement, ethnic minority, health education, malaria, performance arts

## Abstract

**Background:**

In Siem Pang, northeastern Cambodia, malaria transmission persists in remote forested areas populated by ethnic minorities. Engaging affected communities in health education campaigns is challenging due to language, access and literacy constraints. During 2018, a newly established medical research station conducted a health education programme in local villages harnessing traditional songs, arts and crafts, along with theatre, comedy and health talks and quizzes. Health education topics were proposed by community leaders and focused on maternal and child health and malaria. This article describes a process evaluation of these activities, the community's response and whether this was an appropriate form of health education in this context.

**Methods:**

In-depth interviews were conducted with community members, leaders and performers. Interviews were audio-recorded, transcribed and translated to English for thematic analysis.

**Results:**

In total, 65 interviews were conducted; 20 of these were follow-up interviews with respondents interviewed prior to the performances. Respondents were able to recall the key health messages about malaria, antenatal care and infant vaccination. They also showed good awareness of malaria transmission and prevention and described how they enjoyed the events and appreciated the efforts of the project team.

**Conclusions:**

In isolated communities in Cambodia, a health education programme harnessing performance and arts engaged the whole community and its messages were readily recalled and prompted reflection.

## Introduction

Cambodia has seen notable improvements in health outcomes in recent decades and, along with neighbouring countries in the Greater Mekong Subregion (GMS), a large decline in malaria incidence and related mortality.^1^ More recently, progress in Cambodia has stalled, with malaria incidence rising between 2016 and 2018^2^ and the mortality of mothers and children ≤5 years of age continues to remain higher than that of neighbouring countries.^3^^,^^4^ The most recent Cambodia Demographic and Health Survey (CDHS) in 2014 highlighted that only 76% of women received four or more antenatal care visits from a health professional during the course of their last pregnancy and that there had been a slight decline from 79% (CDHS 2010) to 73% of children 12–23 months of age who were fully vaccinated.^5^ There are also notable rural–urban differences in maternal and child health across the country^6^^,^^7^ and the majority of malaria cases now occur in remote forested villages.^2^

In Cambodia and elsewhere, remote ethnic and migrant communities often have lower levels of health literacy and access to treatment and consequently poor health outcomes.^8^ Access, language and literacy constraints present barriers to effectively reaching these communities with health education campaigns.^6^^,^^9^^,^^10^ Drama and performance are alternatives to more didactic approaches and have the potential to effectively reach these isolated communities.^11^^–^^14^ Such programmes have demonstrated some success in terms of increasing health-related knowledge,^15^ for example, regarding human immunodeficiency virus and tuberculosis.^16^ However, questions remain about the role that drama- and arts-based approaches can play in health education programmes.^15^

Drawing on interviews with community members/leaders and drama performers, this article describes a process evaluation of a health education and community engagement programme that harnessed theatre and arts in northeastern Cambodia. The central research question focused on whether this was an appropriate form of health education in this context, the health messages that participants and communities could recall and their opinions of the overall approach and its various elements.

### The drama and arts programme

The drama and arts programme took place in 10 villages across Siem Pang District during 2018. From the planning stage onwards, all aspects of the activity were conducted as a collaboration between local health staff, community leaders and the community engagement team. The community engagement team was based at a newly established research station housed at the Siem Pang Health Centre, which at the time was also the location for a clinical trial evaluating the efficacy of antimalarial treatments for *Plasmodium falciparum* (ClinicalTrials.gov identifier: NCT03355664). Although a long-standing collaboration exists between the research team and the National Malaria Control Programme of Cambodia, this was the first study to be conducted in Siem Pang District.

The programme aimed to engage the communities in several health domains that were identified as priorities in consultation with local health staff and community leaders. These included promoting awareness of malaria prevention and early treatment, advocating infant vaccination and describing the benefits of antenatal care. The programme also aimed to familiarize local communities with the team from the research station along with their research activities. A further goal was to promote and celebrate local ethnic arts and culture, which community leaders felt was being lost over time.

The arts and drama approach to health promotion is often school-based and is used to address sensitive issues, such as sexual and reproductive health: drama is recognized as a particularly useful approach because it allows students to engage in rehearsals of real life.^15^ Additional reasons for selecting this approach for use in Siem Pang were print media, such as leaflets and posters, for engagement purposes, was not deemed appropriate due to the relatively low literacy rates (compared with urban areas);^17^ isolated communities rarely host large events, and these can potentially attract much of the local population and hence the messages reach a wide audience; and incorporating local performers (and costumes) ensures that the health messages are rooted in the familiar and not decontextualized. The activities built on a previous programme of village drama used in western Cambodia,^12^^–^^14^ which was developed by the community engagement team and a drama group in collaboration with community stakeholders. The programme in Siem Pang was further adapted based on feedback during the first of three stages:

1. Orientation and consultation

During a formal orientation meeting at the Siem Pang Health Centre, the activities, target villages and topics for community engagement were discussed. Approximately 50 key stakeholders attended and the meeting was chaired by the Deputy District Governor of Siem Pang. These stakeholders included commune and village leaders, primary school principals, health centre chiefs, the police chief and village malaria workers. The community engagement team and members of the theatre group^12^^–^^14^ (a professional theatre group experienced in village drama) also attended. Attendees were given the opportunity to ask questions about the proposed community engagement plans. Likewise, the community engagement team had the opportunity to obtain feedback and fine tune the project plan and key messages. This was followed by several informal meetings between the project team and community representatives.

2. Village workshops and performances

**Figure 1.**
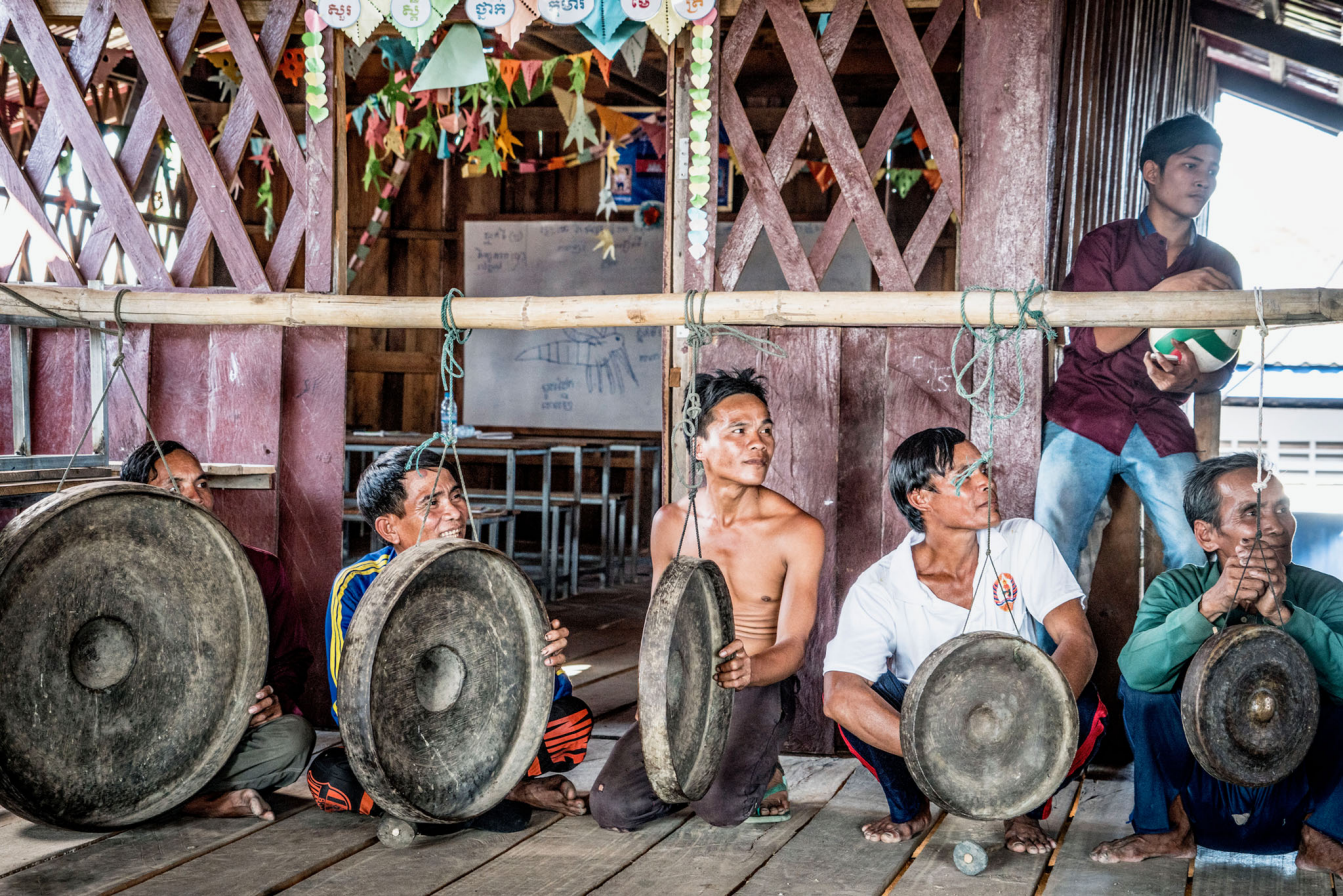
Local villagers rehearsing with their traditional musical instruments in preparation for the village performance.

Subsequently in each target village, the project theatre group conducted art and theatre workshops with village children and young people. Spending 3 days in each village, project staff first met with commune and village leaders, the principal of the local school and village malaria workers to discuss with them the activities and invite the students to participate. With parental consent and school teachers facilitating, students from the area then joined workshops and rehearsals at the school to prepare for a village performance. During rehearsals, performers were instructed on the messages to be conveyed and taught the dialogue and steps. During their time in the villages, project staff invited villagers to talk about their experiences with malaria and show them local costumes, musical instruments, ethnic art and dance (Figure 1). These were then incorporated into the performances.

**Figure 2.**
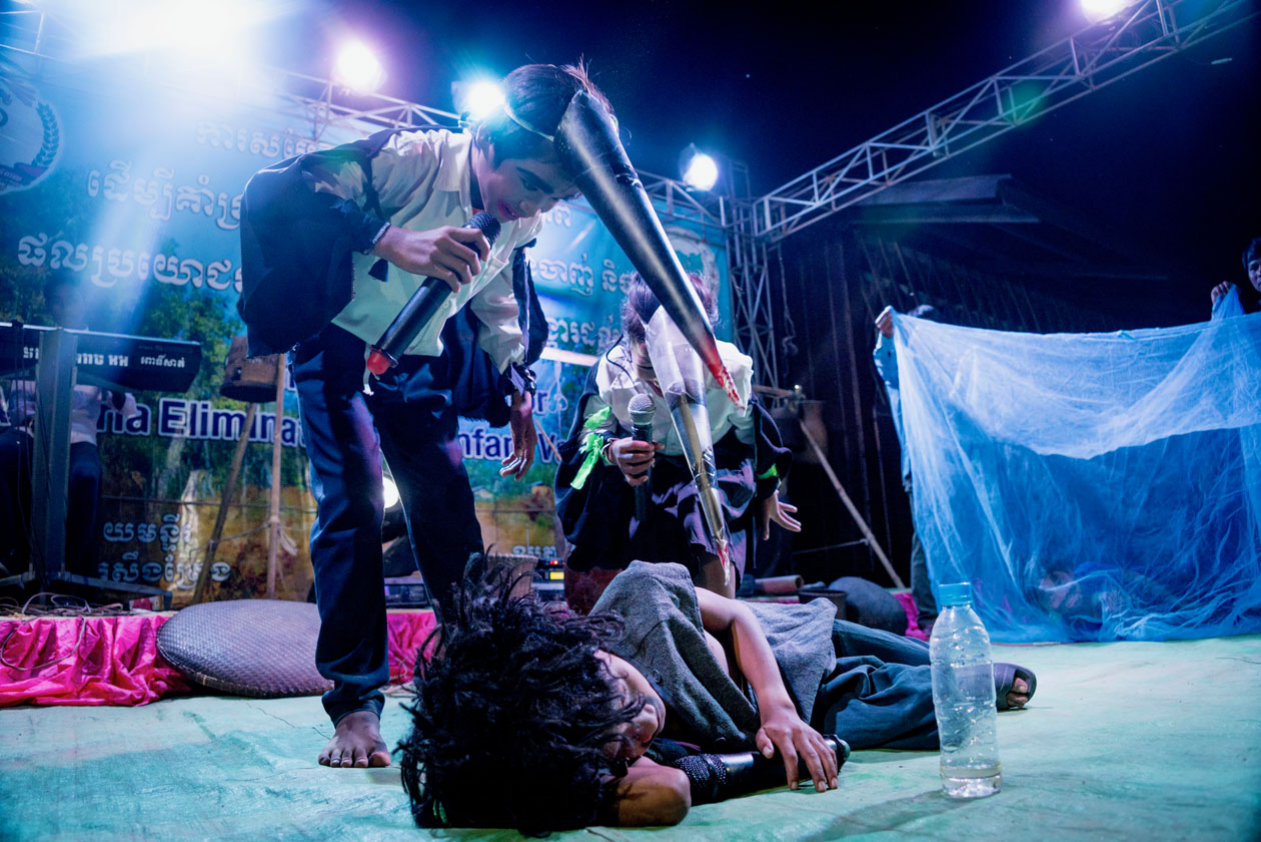
Schoolchildren teaching about malaria prevention during a drama performance—mosquitoes infect (bite) a forest goer with malaria while he sleeps on the ground while his friend (background) remains protected as he sleeps under his bed net.

A stage was constructed in the middle of the village square and the visit of the theatre group to each village culminated on the third evening with local children performing alongside the drama professionals. The evening included games, singing and dancing, health quizzes, a fashion show and a comedy sketch about malaria (Figure 2). All villagers were invited to attend the event. In all villages, the performance was attended by local leaders, village malaria workers and other community members.

3. Closing event

The closing event was held in the town of Siem Pang. The event involved speeches, competitions, performances from professional singers, local traditional dances and an amateur singing competition. Among attendees were leaders of several provincial departments, commune and village leaders, other local institutions and non-governmental organizations (NGOs). The event was broadcast through Reaksmey Hangmeas HD TV. The Deputy Director of the National Center for Parasitology, Entomology and Malaria Control gave a speech. After an initial open audition, selected primary school performers from local communes competed in the singing competition and malaria quizzes, with prizes given to winning performers. Ethnic minority groups from villages previously visited were invited as special guests to perform their local dance and music. 

Photographs and videos of the events were shared on social media (Facebook [https://www.facebook.com/dramaagainstmalaria] and YouTube [https://www.youtube.com/watch?v=h6IOeXaPAmc&t=2s]).

In total, 9365 (69%) villagers either attended or participated in the performances from a target population of 13 610 (Table 1). Some performances attracted people from neighbouring villages and the audience was larger than the population of the target village.

**Table 1.** Number of performers and the populations of the target villages

		Performers, n			Audience, n				
No.	Village	Male	Female	Subtotal	Male	Female	Subtotal	Total participants, n (%)	Total population, N
1	Kham Phouk	17	22	39	200	313	513	552 (67)	820
2	Nhang Sum Thom	18	22	40	308	520	828	868 (68)	1268
3	O Chay	16	18	34	240	300	540	574 (64)	897
4	Khei Nang	22	29	51	300	393	693	744 (185)^b^	402
5	Teak Team	20	27	47	382	500	882	929 (207)^b^	448
6	Khei Svay	16	22	38	498	713	1211	1249 (103)^b^	1212
7	Samor Khnong	19	16	35	213	300	513	548 (64)	856
8	Ban Moung	20	21	41	352	398	750	791 (101)^b^	782
9	Kachanh Teuk	17	24	41	200	259	459	500 (64)	780
10	Siem Pang town^a^	70	87	157	1132	1321	2453	2610 (159)^b^	1640
Total		235	288	523	3825	5017	8842	9365 (69)	13 610

a Closing event.

b Participant numbers exceeded the target village population as a result of people attending the performances from neighbouring villages.

## Methods

To evaluate the process of health education and community engagement, qualitative research methods were used to elicit the perspectives of various stakeholder groups on the arts and drama–based programme. Information from these interviews was combined with monitoring data collected during the engagement. These include attendance figures and logs kept by the engagement team in the form of notes addressing challenges and lessons learned during the programme.

### Setting

**Figure 3.**
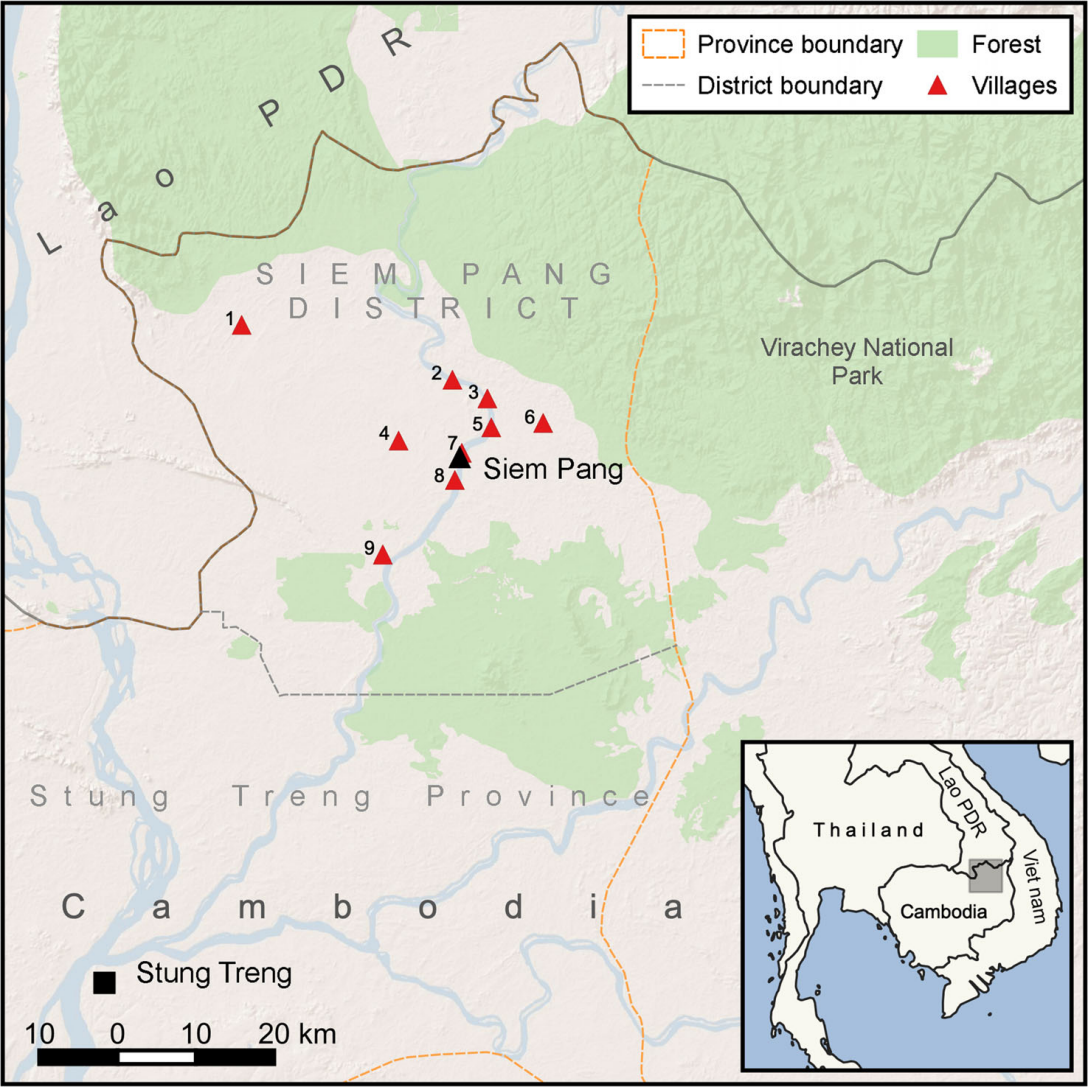
Map of Siem Pang District showing the locations of the target villages: 1. Kham Phouk, 2. Nhang Sum Thom, 3. O Chay, 4. Khei Svay, 5. Teak Team, 6. Khei Nang, 7. Samor Khnong, 8. Ban Moung, 9. Kachanh Teuk and Siem Pang town.

**Figure 4.**
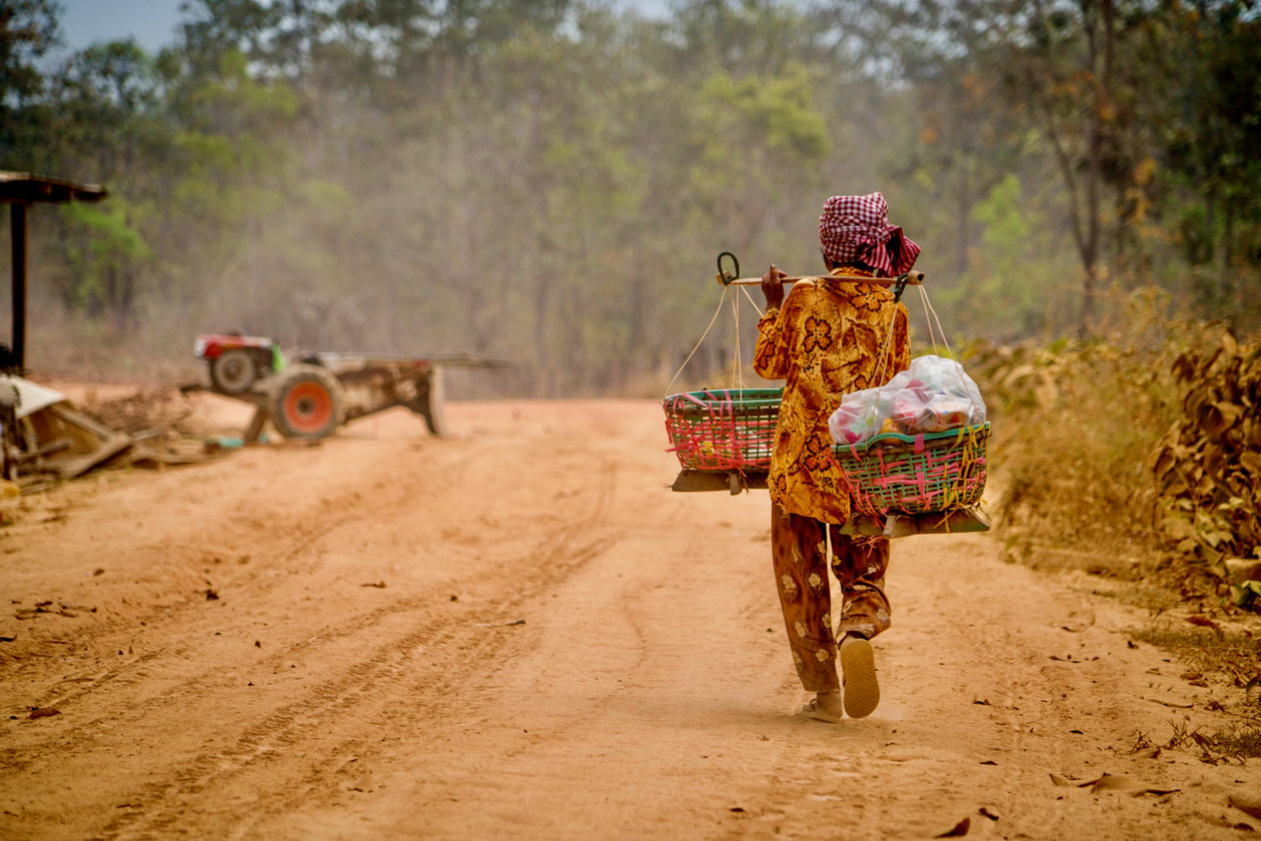
In the rural forest fringe villages of Siem Pang District, livelihoods revolve around subsistence agriculture and the use of forest resources.

Located in northeastern Cambodia, Siem Pang District lies within Stung Treng Province and borders Lao People's Democratic Republic to its north and west. Stung Treng Province, bisected by the Sekong River, is predominantly rural and has among the highest incidence rates of malaria in Cambodia.^2^^,^^18^ Accounting for almost 40% of all malaria cases in Stung Treng, Siem Pang District is a particularly high incidence zone for malaria.^18^ Siem Pang lies on the Sekong River, with large forests to the north and limited road access during the main rainy season (Figure 3). It has a population of around 24 000 people distributed across 28 villages. Most people's livelihoods involve subsistence agriculture and the use of forest resources (Figure 4). The population is ethnically diverse and there are low Khmer literacy rates.

Ten villages were selected for the programme of activities. Villages were selected based on malaria incidence and/or particular geographical isolation. These villages are home to Kaviet and Lao populations and the majority are illiterate in Khmer. These selection criteria ensured that villages likely to have been neglected in past health campaigns were included in our current engagement activities.

### Data collection

Three trained field researchers conducted in-depth interviews. The interviews took place in Khmer and, if needed, translation was sought from other study staff members fluent in Laotian or another local language. Interviews were undertaken with community members in the days prior to the village performances (in stage 2 of the programme) and up to 6 weeks after the performances (including follow-up interviews with the same respondents). Additional interviews with community leaders took place in the weeks after the events and included village chiefs, members of the district council, teachers and village malaria workers. Interviews were also conducted with performers involved in the drama and arts activities 1 or 2 days after the event. Respondents were purposefully selected based on their experience with the community engagement activities and their availability. All respondents gave written informed consent prior to interviews.

To assess awareness of key messages and attitudes towards the various activities, semi-structured interview guides were developed to direct the questioning of each respondent group. The interviews included open questions that focused on awareness of malaria; its transmission, prevention and control; previous information campaigns in the area around malaria and other health issues; and experiences and attitudes towards the various community engagement activities. A flexible approach was taken during the interviews and interviewers were encouraged to follow-up on relevant issues that might not have been directly addressed in the interview guides.

The project team recorded attendances at the various community engagement events and kept a log of activities and challenges. This was made available to the study team for background and interpretation of the interviews.

### Data processing and analysis

With the consent of respondents, all interviews were digitally audio-recorded. The audio files were transcribed and translated into English by a professional transcription and translation service. All transcripts were checked for accuracy by a bilingual (Khmer–English) member of the research team who conducted the interviews. Personal information was removed from the transcripts to maintain confidentiality and protect respondents’ privacy.

The transcripts were coded line by line in NVivo version 11 (QRS International, Melbourne, VC, Australia) using a codebook. At first, codes were based on initial research questions (deductive) and new codes were added in response to emerging themes (inductive). Initial research questions included: What were stakeholder opinions on using drama and arts for community engagement? What health messages did participants recall?

## Results

### Respondents

In the days leading up to the village performances, 20 interviews were undertaken with community members and a further 29 took place up to 6 weeks after the performances. Twenty of these were follow-up interviews with respondents interviewed prior to the performances. A further 11 interviews were conducted with community leaders in the weeks following the event (plus 1 singer). Respondents included village chiefs, members of the district council, teachers and village malaria workers. Interviews were also conducted with five performers involved in the drama and art activities 1 or 2 days after the event.

### Awareness and understanding of the health messages

In the interviews prior to the engagement programme, respondents demonstrated a basic understanding of malaria transmission and its prevention and control. Mosquito bites were recognized as the ‘cause’ of the disease. People knew mosquito bites can be avoided by using bed nets, and some mentioned wearing long-sleeved clothes to prevent bites. Visits to forested areas were seen as increasing the risk of malaria infection because of the high number of mosquitoes in these places. A range of symptoms were linked to malaria, including fever, chills, muscle/generalized aches, headache, malaise, fatigue, dizziness and a metallic taste. Respondents generally knew to visit a village malaria worker or a health facility for malaria treatment. There were also references to self-management of fever using antipyretics.

When interviewed after the events, respondents generally recalled the specific health messages from performances, for example, using insecticide-treated bed or hammock nets to prevent mosquito bites and malaria, ensuring that the nets are the insecticide-treated type (not the untreated type sold in the markets) and not damaged, seeking assistance from village malaria workers, visiting health facilities for pregnancy monitoring as part of antenatal care and following the infant vaccination schedule at health facilities to prevent childhood diseases. Precise details of some of the messages conveyed in the performances were less easily readily restated.

*I: What did you learn from the performance of the children?*

*R: They told us [how] to eliminate malaria.*

*I: To achieve this, what do we have to do?*

*R: Not sure* *…* *we have to sleep in an impregnated bed net, take the hammock net to the forest and wear long-sleeved clothes.*

*[…]*

*I: What transmits the disease?*

*R: Female Anopheles mosquitoes.*

— Interview with a 25-year-old farmer from O Chay village

*I: What about vaccination? What did you learn from the performance regarding vaccination?*

*R: …* *vaccines for infant?!*

*I: What were the key messages? What do infants need?*

*R: They need to get vaccinated.*

*I: How many times?*

*R: I don't remember.*

*I: What diseases do the vaccines prevent?*

*R: Hepatitis, Polio*

*I: What else?*

*R: …*

*I: There are more diseases.*

*R: Pertussis.*

*I: And?*

*R: I don't remember. There are many more.*

— Interview with a 24-year-old farmer from Nhangsum Thom village

In some cases, those involved also shared what they had learned from the performances with other people beyond the event.

*I told my family that they should sleep in a bed net and when they chat or drink beer, they should wear sleeved clothes.*

— Interview with a performer from O Chay village

### Health messaging and behaviour change

Although aware of ways of preventing malaria, respondents described how community members did not always use them. Bed nets were described as uncomfortably hot at night. Respondents noted that the issues that the performances addressed, such as people not using mosquito nets when visiting the forest, were particularly relevant. Respondents were also positive about the impact of the health messages delivered as part of the performances. Despite the linguistic diversity in the villages, because the performance incorporated non-verbal communication (combined with Khmer), the community members generally understood the messages. There was also some optimism about their impact in terms of fostering behaviour change among community members.

*I: Do you believe they will change* *—* *the bad habit of not sleeping in bed nets?*

*R: Yes, I believe they'll change.*

— Interview with a 35-year-old community member (farmer) from Sre Sambo village

Respondents also recognized that some issues could not necessarily be addressed through the delivery of health messages. The nets would still be uncomfortable and mosquito bites were also seen as likely to occur outside of hammock nets when people are working or defecating at night. Encouraging people to seek assistance from village malaria workers or health centre staff would not overcome the limited availability of providers, which sometimes led people to attend private clinics.

### Attitudes towards the arts and drama

The overarching theme from the interviews was the sense of enjoyment that participants associated with the arts and drama events. The enjoyment was linked to the lessons that they had learned about preventing ill health in their communities; the amusing nature of the performances; sharing the events with their own children/grandchildren, nieces or nephews; and seeing local (Khmer traditional and ethnic minority) dance and costumes. Respondents described previous health education activities undertaken by NGOs in their villages but portrayed these events as being very different: this was the first time that they had seen dance and performance combined with messages about health and disease.

*I: What is your opinion of the performance?*

*R: It was exciting that we all want to see it again. And the people could learn about malaria as well as the environment* *.*

— Interview with a 25-year-old community member after the performance in Ban Moung village

The community leaders were particularly happy with the involvement of local children. Minor complaints centred on the lack of time to prepare for the performance and some language difficulties that the younger performers encountered. However, incorporating Kaviet dance and costume was viewed as an effective way of fostering enthusiasm and participation.

*I: What were the performance activities you remember?*

*R: The activities I was interested in were ethnic minority Kong or the harvest dance because they are about to be forgotten and [have been] abandoned for [a] long time.*

— Interview with Siem Pang community leader (district leader)

Respondents who participated in the events described excitement at being involved. For most, this was the first time that they performed in public and hence they reported minor worries; for example, like getting the dance steps right. There was, however, a general desire to repeat the events and to perform again. Several community leaders also mentioned the broader benefits of young people performing in public; for example, gaining in confidence and gaining exposure to their local ethnic traditions through learning about the dance routines and local costumes.

## Discussion

During the drama and arts programme, attendance was high in all villages, which included audience members from outside the immediate area. From the interviews, it appears that community members and performers recalled many of the health messages conveyed in the programme, although some of the details were less easily recounted. Even before the performance, respondents were aware of the fundamentals of malaria prevention and control, which was reinforced through the programme. Respondents recognized the limits of the impact of such a programme, with its potential influence on behaviour conditioned by a range of factors, such as the availability of health services and the nature of forest work (that exposes people to mosquitoes). There was an overall appreciation of using arts and drama to engage communities and offer health education; respondents were particularly positive about the use of local costumes and art.

### Assessing the impact of drama and arts

As recently reported among forest-goers in Siem Pang,^19^ there was a relatively high awareness of malaria transmission and prevention. This awareness made it even more difficult to discern the impact of some malaria-related health messages. The responses indicate, however, that community members were able to recall many of the specific health messages that were incorporated into the performance. A different study design, such as a trial combined with representative surveys, would have enabled a more comprehensive assessment of the impact of the arts and drama approach for uptake of the health messages. In this instance, resource limitations meant that such an approach was not feasible, but it should be considered in the future.

### Tailoring arts and drama to promote engagement

Engagement and health education programmes have incorporated theatre because of its capacity to examine health and social problems, raise awareness, reach decision makers, promote activism and disseminate study findings.^20^ The activities in Siem Pang were a development of earlier approaches^12^^–^^14^ and were adapted to this different cultural context, with participants from Lao and Kaviet communities. Villagers had the opportunity to showcase their traditional costumes, dance and musical instruments in the public performances. This helped the participants and the audience to gain a sense of ownership of the performances and this element was particularly popular among the local Kaviet and Lao populations.

### Engaging the wider community

Attendance at the 2018 Siem Pang village drama performances was higher than in previous engagement projects in Battambang (2016) and Pailin (2017).^13^^,^^14^ As the respondents mentioned, the communities in Siem Pang are particularly isolated (more so than the villages that hosted engagement activities in 2016 and 2017) and such activities are rare and therefore are valued by the local population.

This arts and drama programme sought to have an impact beyond the performers directly involved in the performances. Involving students as performers meant that they had to learn lines and routines and generally master the subject matter. However, through community performances, the programme prompted greater involvement from parents, teachers and relatives, who were particularly invested in the performances. The interviews further highlighted how having a stake in the performances and enjoying the performances underpinned engagement.

### Strengths, limitations and lessons for future programmes

Interviewing a range of different respondent types, including those with varying degrees of experience with the activities ensured a breadth of perspective. The interviews provided information on community opinions of the engagement programme and whether the health messages were recalled after the event. For logistical reasons, it was not possible to conduct a questionnaire-based survey to more comprehensively assess any impact of the programme on malaria awareness and knowledge (either through a before-and-after or trial-type design). Prior to commencement there was not sufficient time to visit the target communities and conduct detailed formative research to assess the levels of awareness of the health topics. This limited the tailoring of messages in each village. Future programmes will ideally incorporate this into the planning stage. In light of the popularity of this initial programme of community engagement, future activities are planned and could be linked to sensitization and mobilization for research studies.

## Conclusions

Performance and arts as part of a health information programme was popular in target villages in the Siem Pang District of northeastern Cambodia. The success of the programme in terms of the attendance, engagement and resonance of the health messages was based on incorporating local performance and costumes and involving local students in a community performance. This increased ownership and engagement in the wider community. Early engagement with local stakeholders ensured that key health messages aligned with local health strategies. Performance and arts could play an important role in health information campaigns, particularly in remote areas often neglected as a result of the challenges of linguistic diversity. Further research is needed to more fully assess the uptake of health messages conveyed, potentially using experimental study designs.

## Data Availability

The data on which this article is based cannot be shared publicly because the informed consent obtained from respondents did not specify that data would be made publicly available and the public availability of data would compromise the privacy of respondents. The data are available upon request to the Mahidol Oxford Tropical Medicine Research Unit Data Access Committee (https://www.tropmedres.ac/units/moru-bangkok/bioethics-engagement/data-sharing) complying with the data access policy (https://www.tropmedres.ac/files/moru-bangkok-files/moru_data_sharing_policy_v2-0.docx) for researchers who meet the criteria for access to confidential data.
